# Efficacy and safety of Linggui Zhugan decoction in the treatment of chronic heart failure with Yang deficiency: A protocol for systematic review and meta-analysis

**DOI:** 10.1097/MD.0000000000026012

**Published:** 2021-05-21

**Authors:** Yumeng Li, Huaqin Wu, Bingxuan Zhang, Xia Xu, Yajiao Wang, Qingqiao Song

**Affiliations:** a Department of General Medicine; b Department of Cardiovascular, Guang’anmen Hospital, China Academy of Chinese Medical Sciences, Beijing, China.

**Keywords:** chronic heart failure, Linggui Zhugan decoction, protocol, systematic review

## Abstract

**Background:**

Chronic heart failure (CHF) is an advanced stage of various heart diseases and has become a major global health problem. In 2018, the Chinese guideline for the diagnosis and treatment of HF suggested adding traditional Chinese medicine (TCM) as an adjunct to the treatment of CHF, but also pointed out the need for more convincing clinical evidences. Linggui Zhugan decoction (LGZGD) is one of the widely used TCM for CHF treatment, especially for patients with Yang deficiency. Given that treatment based on syndrome differentiation is an important principle in TCM, we provide a protocol to systematically evaluate efficacy and safety of LGZGD for CHF with Yang deficiency.

**Methods:**

We will search the following electronic databases from inception to April 30, 2021, including PubMed, EMBASE, Cochrane Central Register of Controlled Trials, Chinese National Knowledge Infrastructure, VIP Information Database, Chinese Biomedical Literature Database (CBM), and Wanfang Database. Randomized controlled trials (RCT) examining the LGZGD on CHF patients with Yang deficiency will be included. Study selection, data extraction and quality assessment will be conducted by 2 researchers, respectively. The primary outcome measures will be n-terminal brain natriuretic peptide and left ventricular ejection fraction. The risk of bias will be evaluated by the Cochrane Collaboration tool. We will use the fixed-effects model or random-effects model of RevMan V.5.3 based on the results of heterogeneity assessment. The evidence quality of results will be assessed by Grading of Recommendations Assessment, Development and Evaluation (GRADE) system.

**Results:**

It will provide the results about efficacy and safety of LGZGD in the treatment of CHF with Yang deficiency by various comprehensive assessments.

**Conclusions:**

This study will provide reliable evidences about the efficacy and safety of LGZGD in the treatment of CHF with Yang deficiency.

**Ethics and dissemination:**

Ethical approval is not necessary for this study because the data extracted does not involve any individual privacy. We plan to presented the results of this review in a peer-reviewed journal.

**PROSPERO registration number:**

CRD 42019140797.

## 1 Introduction

Chronic heart failure (CHF) is a complex clinical syndrome characterized by reduced cardiac output, insufficient perfusion of tissues and organs or venous system congestion.^[1]^ With increasing risk factors due to poor lifestyles and longer survival of patients with cardiovascular diseases, HF has become a major global health problem, affecting at least 26 million people all over the world.^[2]^ Recent data shows that there are about 5.7 million people with HF in United States, and it is estimated that more than 8 million people will accompany by HF after 10 years, which means a 46% increase in prevalence from 2012 to 2030.^[3]^ According to the 2018 Report on Cardiovascular Diseases in China, the number of patients suffering from HF are 4.5 million.^[4]^

CHF is a serious condition with typical symptoms including breathlessness, swollen limbs and fatigue.^[1]^ In addition, CHF is characterized by decreased exercise tolerance mainly related with skeletal muscle atrophy and dysfunction, which limits daily living activities and reduces health-related quality of life (QoL).^[5]^ In spite of the current evidence-based medications or devices have provided benefits for millions of patients with CHF, rehospitalization rates and 5-year mortality remain high.^[3,6]^ It is reported that the 5-year death rate of people with HF is more than 50% in US.^[7]^ Among Chinese in Hong Kong, all-cause mortality of patients with new-onset HF is 19.5% at 1 year, 32.1% at 2 years, and 54% at 5 years.^[8]^ These therapeutic interventions also impose a huge economic burden. In 2012, the estimated health expenditure for HF was $30.7 billion in the US, with 68% was attributable to direct medical costs.^[9]^ Furthermore, long-term use of the referred drugs may be accompanied by side effects, such as electrolyte disturbance and hypotension.^[10,11]^

It highlights the need for research more potential treatments of CHF to improve functional capacity, prevent hospitalization and prolong life. Traditional Chinese medicine (TCM) is increasingly used to treat CHF in China, showing some advantages in reducing costs, enhancing physical condition and improving prognosis.^[12]^ Treatment based on syndrome differentiation is an important principle in TCM clinical practice. The concept of syndrome is unique to TCM, which is mainly evaluated by 4 traditional diagnostic methods including inspection, listening and smelling, inquiry, and palpation. It is a summary of the pathological status, which reflects stages of disease and individual reaction of patients.^[13]^ Therefore, TCM prescriptions that corresponds to the syndromes can significantly improve the clinical efficacy.

According to TCM theory, the syndromes of CHF show a regular evolution. Initially, there are deficiencies of heart-qi and heart-yang, accompanied by blood stasis. With the development of the disease, the spleen-yang is damaged and fluid cannot be transported, causing the phlegm-turbid stagnation. In the later stage, deficiency of kidney-yang occurs, leading to the fluid retention. Consequently, the pathogenesis of heart failure in TCM can be summarized as internal deficiency, blood stasis and fluid retention, while Yang deficiency is the fundamental pathogenesis.^[14]^ Therefore, the treatment of CHF with warming yang and resolving fluid-retention methods are widely used in clinical practices.

Linggui Zhugan decoction (LGZGD) is one of the representative prescriptions, which comes out of Treatise on Febrile Diseases by Zhang Zhongjing. It consists of Fuling (poria cocos), Guizhi (cassia twig), Baizhu (atractylodes) and Gancao (licorice). Experimental studies indicated that LDZGD improved myocardial tissue injury and resisted ventricular remodeling by inhibiting the inflammatory response in HF.^[15]^ It also delayed progression of disease by inhibiting the overactivation of renin-angiotensin-aldosterone system in rats of CHF.^[16]^ Network pharmacological study demonstrated that LGZGD improved CHF may be related to the regulation of renin-angiotensin-aldosterone system and sympathetic nervous system.^[17]^

There are also some systematic reviews involving randomized controlled trials (RCTs) of LGZGD, which suggesting that efficacy of LGZGD combining with western medicine is better than western medicine alone.^[18–20]^ However, these studies have some limitations. Firstly, inadequate power attribute to limited sample size or poor quality of the included literatures. Next, most of them adopted the total effective rate, a subjective outcome measure, as the primary outcome, which affects reliability of the results. Moreover, these reports did not take into account the TCM syndromes of the included patients, which could not reflect the principle of treatment based on syndromes differentiation. Therefore, consider the combination of disease and syndrome, the aim of this study is to evaluate the efficacy and safety of LGZGD for CHF patients with Yang deficiency by conducting a systematic review and meta-analysis.

## 2 Methods

### 2.1 Protocol registration

This protocol is established according to the Preferred Reporting Items for Systematic Reviews and Meta-Analyses Protocols (PRISMA-P) statement guidelines^[21]^ and has been registered on the International Prospective Register of Systematic Reviews (PROSPERO) (registration number: CRD 42019140797).

### 2.2 Inclusion criteria

#### 2.2.1 Study designs

RCTs evaluating the efficacy and safety of LGZGD on CHF patients with Yang deficiency will be included, regardless of blinding. Only human studies will be accepted in this review.

#### 2.2.2 Participants

Only studies that specifically referred to the participants (18 years or older) as having CHF will be included, regardless of types of CHF (i.e., preserved, moderately reduced and reduced ejection fraction). Meanwhile, patients should meet the description of Yang deficiency syndrome in clinic terminology of TCM diagnosis and treatment (GB/T 16751. 2- 1997).^[22]^

#### 2.2.3 Interventions

The experimental group should combine conventional medical treatment with LGZGD as intervention measures, regardless of dosages and formulations. While the control group should receive the same conventional medical treatment alone or combine with placebo. Conventional medical treatment should meet the recommendations of 2016 ESC guidelines.^[1]^ Treatment durations of both groups should be at least two-week.

#### 2.2.4 Outcomes

The primary outcome measures will be n-terminal brain natriuretic peptide (NT-proBNP) and left ventricular ejection fraction (LVEF). The secondary outcome measures include clinical total effective rate, 6-minutes walking test, rehospitalization rates or mortality, quality of life (QoL) and adverse events. Clinical total effective rate will be assessed by changes of New York Heart Association function classification. QoL will be assessed by Minnesota Living with Heart failure questionnaire.^[23]^

### 2.3 Exclusion criteria

Studies with following conditions will be excluded: (a) non-RCTs, including case report, reviews, editorials and cell or animal experimental studies; (b) either the experimental or control group contains other complementary and alternative therapies, such as Chinese herbal medicines, acupuncture, Tai Chi and so on; (c) absence of quantitative outcome measures; (d) duplication reporting the same results.

### 2.4 Data sources

#### 2.4.1 Electronic searches

We will carry out a systematic search in PubMed, EMBASE, Cochrane Central Register of Controlled Trials, Chinese National Knowledge Infrastructure (CNKI), VIP Information Database, Chinese Biomedical Literature Database (CBM), and Wanfang Database. The retrieval time is from their inception up to April 30, 2021. The search strategies of PubMed database are shown in Table 1 and will be modified to suit other databases. We will include articles reported in the Chinese and English without restriction on publication status.

**Table 1:** Search strategy sample of MEDLINE.

Number	Search terms
#1	“heart failure”[MeSH Terms] OR “cardiac failure”[Title/Abstract] OR “heart decompensation”[Title/Abstract] OR “decompensation heart”[Title/Abstract] OR “myocardial failure”[Title/Abstract] OR “congestive heart failure”[Title/Abstract] OR “heart failure congestive”[Title/Abstract] OR “diastolic heart failure”[Title/Abstract] OR “cardiac insufficiency”[Title/Abstract] OR “left ventricular dysfunction”[Title/Abstract]
#2	“yang deficiency”[Title/Abstract] OR “Yangxu”[Title/Abstract]
#3	#1 AND #2
#4	“Linggui Zhugan formules”[Title/Abstract] OR “lingguizhugan decoction”[Title/Abstract]
#5	“clinical trials as topic”[MeSH Terms] OR “clinical trial”[Publication Type] OR (“clinical”[Title/Abstract] AND “trial”[Title/Abstract]) OR “random∗”[Title/Abstract] OR “random allocation”[MeSH Terms]) NOT ((“animals”[MeSH Terms:noexp] OR “animals”[All Fields]) NOT “humans”[Title/Abstract]
#6	# 3 AND #4 AND #5

#### 2.4.2 Additional search

The electronic database search will be supplemented by searching National Institutes of Health (NIH) clinical registry Clinical Trials.gov, International Clinical Trials Registry Platform (ICTRP) and Chinese Clinical Trial Registry. Furthermore, the reference lists of included studies and relevant reviews will be manually searched to identify other potential literatures.

### 2.5 Study selection and data extraction

#### 2.5.1 Selection of studies

**Figure 1:**
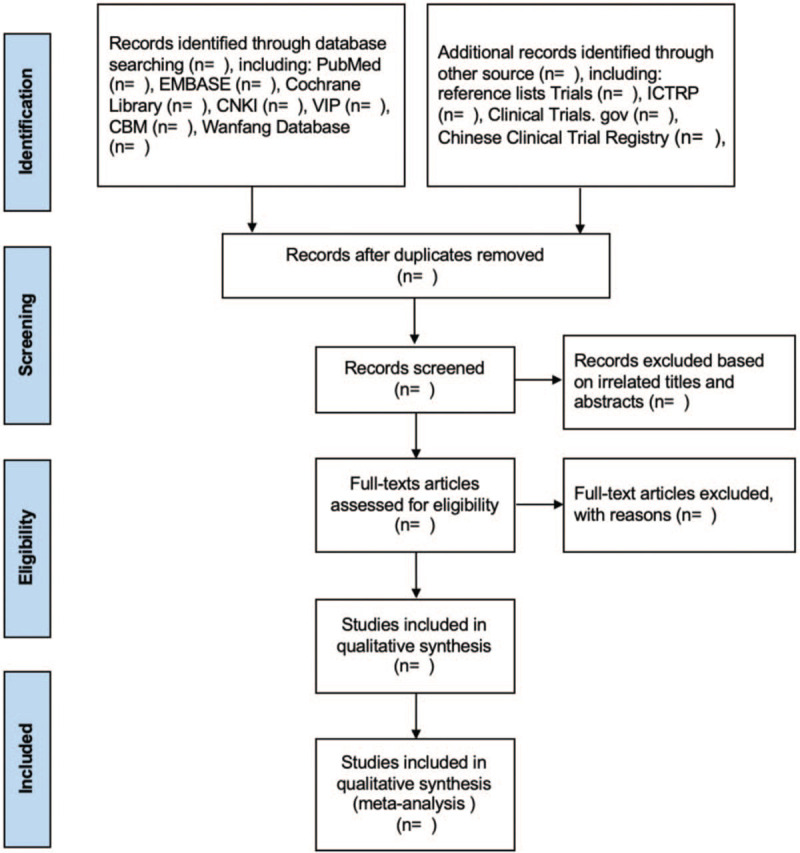
A PRISMA flow diagram of the literature screening and selection processes.

NoteExpress Version 3.2 will be used for literatures management. Firstly, duplicate documents will be deleted by software. And then 2 reviewers (YL and YW) will remove irrelevant articles independently by screening the titles and abstracts. If the reports meeting the predefined eligibility criteria or which there is any uncertainty, we will obtain and read full texts. All the reasons for excluding trials will be recorded. A Preferred Reporting Items for Systematic Reviews and Meta-analysis flow chart will be drawn to illustrate the study selection procedure (Fig. 1).

#### 2.5.2 Data extraction and management

Based on the predefined data extraction forms, the data will be extracted independently by 2 reviewers (YL and XX). Including: (a) general information of included trials, including the first author's name, study publication year and study design; (b) characteristics of included patients: gender age, previous medication, sample size, drop-outs and reasons; (c) interventions: interventions of experimental and control groups, treatment frequency and duration, dosage of western medicine; (d) outcome measures: NT-proBNP, LVEF, clinical total effective rate, 6-MWT, rehospitalization rates, mortality, QoL and adverse events. Two reviewers will resolve disagreements about study selection and data extraction by discussion and a third participant (HW) will adjudicate unresolved questions.

#### 2.5.3 Risk of bias in the included studies

Two reviewers (YL and BZ) will independently evaluate the risk of bias for each study by using the Cochrane Collaboration tool,^[24]^ including random sequence generation, allocation concealment, blinding, incomplete outcome data, selective outcome reporting, and other potential sources of bias. Each domain was ranked as “low risk of bias,” “high risk of bias” or “unclear risk of bias”. Disagreements will be resolved first by discussion and then by consulting a third participant (HW) for arbitration.

#### 2.5.4 Measures of treatment effect

For continuous outcomes, mean difference (MD) will be used as a summary statistic if data measured with same scale, otherwise standardized mean difference (SMD) will be used. For dichotomous outcomes, risk ratio (RR) will be used in the meta-analysis. All of these data will be summarized with a 95% confidence interval (CI).

#### 2.5.5 Dealing with missing data

When there are missing data, we will attempt to contact with the original authors via email or telephone to obtain the relevant information. If absent data cannot be obtained, we will still conduct an analysis using available data and assess the possible impact of missing data on the results by sensitivity analysis.

#### 2.5.6 Assessment of heterogeneity

Statistical heterogeneity between the studies will be assessed using the *x*^2^ test and *I*^2^ statistic. According to the Cochrane Handbook for Systematic Reviews of Interventions, ^[25]^*P* < .1 of *x*^2^ or *I*^2^ ≥ 50% indicates high levels of heterogeneity, and then we will try to perform subgroup analysis to explore clinical or methodological heterogeneity.

#### 2.5.7 Data synthesis and meta-analysis

Review Manager software (RevMan Version 5.3, Copenhagen: The Nordic Cochrane Center, The Cochrane Collaboration, 2014) will be used to combine and calculate the outcomes. We will decide whether to perform meta-analysis and which model to use based on the results of heterogeneity assessment. If the heterogeneity is not significant, fixed effect model will be conducted. If heterogeneity is observed (*P* < .1 of *x*^2^ or *I*^2^ ≥ 50%), the random effect model will be chosen. A narrative synthesis will be performed for the studies that could not be quantitatively pooled.

#### 2.5.8 Subgroup analysis

If a sufficient number of studies are included, we will carry out subgroup analyses to explore possible reasons underlying the heterogeneity based on the follows: characteristics of participants, control drugs, treatment durations and follow-up period.

#### 2.5.9 Sensitivity analysis

In order to evaluate the stability of the results, sensitivity analyses will be performed by removing the studies with high risk of bias or missing data.

#### 2.5.10 Assessment of publication bias

To determine whether reporting bias is present, publication bias will be assessed by funnel plot if sufficient number of trials (more than 10) included.

#### 2.5.11 Assessment of evidence quality

We plan to evaluate the evidence quality of results in this systematic review by using guidelines of the Grading of Recommendations Assessment, Development and Evaluation (GRADE).^[26]^ The quality of evidence will be assessed across the domains of risk of bias, consistency, directness, precision and publication bias. Level of evidence quality is classified into 4 types: high, moderate, low or very low. Level of recommendation is divided into 2 types: strong recommendation and weak recommendation.

## 3 Discussion

CHF is an advanced stage of various heart diseases with high mortality and rehospitalization rates.^[27]^ The goals of treatment in CHF are improving patients’ clinical symptoms, functional capacity and quality of life, preventing hospital admission and reducing mortality.^[1]^ Previous researches have demonstrated that integrative medicine therapy using TCM combined with western medicine possibly had a better effect in patients with CHF.^[28]^ In 2018, the Chinese guidelines for the diagnosis and treatment of heart failure also suggest adding traditional Chinese medicine (TCM) as an adjunct to the treatment of heart failure, but also point out the need for convincing clinical evidence. It also emphasized the need to pay attention to the potential adverse reactions of integrated traditional Chinese and Western medicine treatment.^[27]^

Treating patients based on syndrome differentiation is an important aspect of TCM. LGZGD is one of the commonly used TCM in the treatment of CHF, especially for patients with Yang deficiency. Clinical trials have shown that LGZGD combined with Western medicine treatment can better improve heart function indicators, such as B-type brain natriuretic peptide (BNP) and LVEF, and promote the quality of life in CHF patients with Yang deficiency.^[29,30]^ However, the previous systematic reviews of clinical trials for LGZGD in the treatment of CHF did not focus on the syndromes of the subjects, which fails to reflect the characteristics and advantages of TCM treatment.

Given these circumstances, we displayed this protocol for a systematic review and meta-analysis of providing up-to-date data to appraise the efficacy and safety of LGZGD for CHF patients with Yang deficiency. It will be conducive to provide more reliable evidence for the treatment of CHF with TCM and make recommendations for clinical trials in the future.

## Author contributions

**Conceptualization:** Yumeng Li, Qingqiao Song.

**Data curation:** Huaqin Wu, Bingxuan Zhang.

**Formal analysis:** Yumeng Li, Yajiao Wang, Qingqiao Song.

**Funding acquisition:** Qingqiao Song, Bingxuan Zhang, Yumeng Li.

**Methodology:** Yumeng Li, Bingxuan Zhang.

**Project administration:** Qingqiao Song, Huaqin Wu.

**Resources:** Yumeng Li, Bingxuan Zhang, Qingqiao Song.

**Software:** Yumeng Li, Xia Xu, Yajiao Wang.

**Supervision:** Qingqiao Song, Huaqin Wu.

**Validation:** Yumeng Li, Huaqin Wu, Bingxuan Zhang, Xia Xu, Yajiao Wang, Qingqiao Song.

**Visualization:** Yumeng Li, Yajiao Wang, Bingxuan Zhang.

**Writing – original draft:** Yumeng Li, Xia Xu, Yajiao Wang.

**Writing – review & editing:** Qingqiao Song, Huaqin Wu, Bingxuan Zhang.
